# Antioxidant Activity of Hemp (*Cannabis sativa* L.) Seed Oil in *Drosophila melanogaster* Larvae under Non-Stress and H_2_O_2_-Induced Oxidative Stress Conditions

**DOI:** 10.3390/antiox10060830

**Published:** 2021-05-22

**Authors:** Jelena Vitorović, Nataša Joković, Niko Radulović, Tatjana Mihajilov-Krstev, Vladimir J. Cvetković, Nikola Jovanović, Tatjana Mitrović, Ana Aleksić, Nemanja Stanković, Nirit Bernstein

**Affiliations:** 1 Department of Biology and Ecology, Faculty of Sciences and Mathematics, University of Niš, 18000 Niš, Serbia; jelena.rajkovic@pmf.edu.rs (J.V.); natasa.jokovic@pmf.edu.rs (N.J.); tatjana.mihajilov-krstev@pmf.edu.rs (T.M.-K.); vladimir.cvetkovic@pmf.edu.rs (V.J.C.); nikola.jovanovic1@pmf.edu.rs (N.J.); tatjana.mitrovic@pmf.edu.rs (T.M.); ana.aleksic@pmf.edu.rs (A.A.); 2 Department of Chemistry, Faculty of Sciences and Mathematics, University of Niš, 18000 Niš, Serbia; niko.radulovic@pmf.edu.rs; 3 Institute of Public Health, 18000 Niš, Serbia; nemanjastanko@yahoo.com; 4 Institute of Soil Water and Environmental Sciences, Volcani Center, Rishon LeZion 15159, Israel

**Keywords:** hemp, seed, hemp oil, chemical analysis, antioxidant activity, H_2_O_2_, oxidative stress, *Drosophilamelanogaster*, larvae, life cycle, antimicrobial

## Abstract

The oil extracted from hemp seeds has significant nutritional and biological properties due to the unique composition of polyunsaturated fatty acids and various antioxidant compounds. The potential of this oil for the prevention of oxidative stress and for the treatment of oxidative-stress-induced ailments is of increasing interest. Most studies of hemp seed oil were conducted in-vitro, meaning we lack information about effects and activity in vivo. In the present study, we evaluated the hypothesis that hemp seed oil at different concentrations improves the oxidative state of *D. melanogaster*, under non-stress as well as hydrogen-peroxide-induced stress. We analyzed the effects of hemp seed oil on oxidative stress markers and on the life cycle of *D.* *melanogaster* under non-stress and hydrogen-peroxide-induced stress conditions. *D.* *melanogaster* larvae were exposed to hemp seed oil concentrations ranging from 12.5 to 125 μL/mL. The results revealed that under non-stress conditions, oil concentrations up to 62.5 µL/mL did not induce negative effects on the life cycle of *D. melanogaster* and maintained the redox status of the larval cells at similar levels to the control level. Under oxidative stress conditions, biochemical parameters were significantly affected and only two oil concentrations, 18.7 and 31.2 µL/mL, provided protection against hydrogen peroxide stress effects. A higher oil concentration (125 μL/mL) exerted negative effects on the oxidative status and increased larval mortality. The tested oil was characterized chemically by NMR, transesterification, and silylation, followed by GC-MS analyses, and was shown to contain polyunsaturated fatty acid triglycerides and low levels of tocopherols. The high levels of linoleic and linolenic acids in the oil are suggested to be responsible for the observed in vivo antioxidant effects. Taken together, the results show that hemp seed oil is effective for reducing oxidative stress at the cellular level, thus supporting the hypothesis. The obtained results point to the potential of hemp seed oil for the prevention and treatment of conditions caused by the action of reactive oxygen species.

## 1. Introduction

Plants are sources of the many natural bioactive compounds, which are of increasing interest for their pharmacological potential including antioxidant, antitumor, anti-inflammatory and antimicrobial activities. Oils extracted from the seeds of various plants have attracted much attention in recent years due to their nutritional and pharmacological properties.

Hemp (*Cannabis sativa* L.) is an annual plant of the Cannabaceae family that is cultivated for a range of purposes, including the production of fiber, shives, secondary metabolites (including cannabinoids), and the nutritionally valuable seeds [1]. The hemp plant contains a rich profile of bioactive phytochemicals, including cannabinoids, terpenes, sugars, steroids, phenols, flavonoids, and nitrogenous compounds [2,3,4]. Of special interest is the composition of the highly nutritional hemp seeds, which are traditionally consumed raw or in the form of oil containing a unique fatty acid profile, which is extracted from the seed by cold pressing [5,6]. The extracted hemp seed oil contains linoleic acid (18:2 omega-6) and α-linolenic acid (18:3 omega-3) at a ratio of 2.5:1–3:1; its consumption is considered to provide a cardioprotective effect and improve the lipid profile. The α-linolenic acid that can be found in hemp seed oil has anti-inflammatory and immunoregulatory activities [7,8], while the profile of fatty acids of hemp seed oil was also demonstrated to have positive effects on skin appearance and function [9]. Antioxidant phytomolecules, such as tocopherols, phenols, polyphenols, and lignanamides in the oil, are important for cell protection against oxidative stress [10,11,12,13].

Since oxidative stress has been etiologically implicated in a wide range of medical conditions [14] and its treatment has been proven to positively affect the course and progression of diverse disease states, different plants have been widely investigated for their potential as antioxidants. Reactive oxygen species are produced in cells as part of the normal physiological and metabolic processes, and cells have universal antioxidative protective mechanisms that maintain redox balance [15,16]. Under stress conditions, the balance between reactive species production and antioxidant defense is disturbed and the result is the development of oxidative stress. Under oxidative stress, antioxidants from foods, plants, or in the form of nutraceuticals or pharmaceutical supplements may enhance the antioxidant defense and help reduce the condition of stress. Antioxidants scavenge and neutralize free radicals or strengthen the innate antioxidative ability of cells acting on existing antioxidant protection systems [17].

Few studies have investigated the potential healing effects of hemp seeds or hemp-seed-derived products on cardiovascular and neurodegenerative diseases associated with oxidative stress in animal models [18,19,20]. The results indicate that the antioxidative effects of hemp seed food supplementation can improve the redox status under certain conditions. Jurgoński et al. [21] showed that native and partially defatted hemp seeds improved the antioxidant status in the liver of diet-induced obese rats.

The biological activities of hemp seed oil, and especially the effects on the oxidative state under non-stress and oxidative-stress conditions, have been poorly studied in animal models, despite the unique chemical composition. The accumulated results reveal the antioxidant potential of the oil [22,23,24,25,26,27,28]. Fotschki et al. [29] reported that hemp seed oil induced oxidative stress in livers of obese rats, while in healthy rats it positively affected the antioxidant status. The use of whey-derived proteins enriched with hemp seed oil in the rats’ diet led to a reduction of oxidative stress marker in post-ischemic heart samples [30]. Toxic effects of copper in fish were overcome by application of dietary hemp seed oil, which was explained by a prooxidant–antioxidant balance at ω-3 and ω-6 fatty acid levels [31]. However, the high content of polyunsaturated fatty acids that are susceptible to lipid peroxidation [32,33] can prevent the use of the oil for oxidative-stress-associated diseases. It is, therefore, important to examine the effects of hemp seed oil on the cell redox status under (normal) basal conditions (to judge its potential as a preventative treatment), as well as its application for the reduction of chronic oxidative stress. This dual assessment will reveal possible beneficial or harmful effects of the oil under different conditions.

The aim of this study was, therefore, to examine the in vivo biological activities of hemp seed oil related to its antioxidative function under normal and H_2_O_2_-induced oxidative stress conditions. The hypothesis guiding the workplan was that hemp seed oil at specific concentrations improve the oxidative state of *D. melanogaster*, under non-stress as well as hydrogen-peroxide-induced stress conditions. *Drosophila melanogaster* larvae were used as the biological model system for the study.

## 2. Materials and Methods

### 2.1. Chemicals

All chemicals were purchased from Sigma Chemical Co. (St. Louis, MO, USA). The hemp oil used for the study is a commercial preparation of cold-pressed hemp seed oil (*Cannabis sativa* L.) (World of hemp, Kisač, Serbia).

### 2.2. Chemical Analysis

#### 2.2.1. NMR

The proton (^1^H) NMR spectrum (with ^13^C decoupling and sufficient FID accumulation to allow a satisfactory signal-noise ratio) was recorded on a Bruker Avance III 400 spectrometer (Bruker Corporation, Fällanden, Switzerland) operating at 400 MHz. The solution was prepared in CDCl_3_ with chemical shifts (in ppm) referenced to tetramethylsilane.

#### 2.2.2. GC-MS Analyses

GC-MS analyses were performed on a Hewlett Packard 6890N gas chromatograph equipped with a DB-5MS fused silica capillary column (5% phenylmethylsiloxane, 30 m × 0.25 mm, film thickness 0.25 μm; Agilent Technologies, Santa Clara, CA, USA) and coupled with a 5975B mass selective detector from the same company. The injector and interface were operated at 250 and 320 °C, respectively. The following temperature program was used: oven temperature was raised from 70 to 315 °C at a heating rate of 5 °C/min and then isothermally held for 30 min. As a carrier gas, helium was used with a flow of 1.0 mL/min. Regarding the samples, 1 μL of the sample solution in diethyl ether or pyridine (10 mg dissolved in 1 mL) was injected in a split mode (split ratio 40:1). The mass selective detector was operated at the ionization energy of 70 eV, in the 35−850 amu range, and with a scanning speed of 0.34 s. The relative abundance of the detected constituents was calculated from peak areas without the use of correction factors.

Silylation of the samples: A sample (10 mg) of hemp seed oil was placed into a GC vial. Afterward, 100 µL of pyridine, 100 µL of *N*-methyl-*N*-(trimethylsilyl)trifluoroacetamide, and one drop of trimethylsilyl chloride were added. The vial was capped and heated for 1 h at 60 °C in a heating block. After cooling to room temperature, 1 µL of the pyridine solution of TMS derivatives was injected. The GC-MS program used for this purpose was identical (70(0′)/5 °C min^−1^/315(30′)) to the one used to record the original sample prior to silylation, but also included a 7 min delay (the time that elapsed after the injection until the MS detector turn on).

Methanolysis of the samples: Here, 100 mg of metallic Na was dissolved in anhydrous methanol (20 mL), cooled to room temperature, then 250 mg of hemp seed oil dissolved in MeOH was added under stirring, brought to reflux, and quenched with ice water. This was followed by an immediate extraction of the reaction mixture with diethyl ether (3 × 20 mL). The combined organic layers were washed with brine, dried with anhydrous MgSO_4_, then the solvent was removed in vacuo. The methyl esters were analyzed by GC-MS as described above.

### 2.3. In Vivo Analysis of the Effects of Hemp Seed Oil on Oxidative Stress Markers and the Life Cycle of D. melanogaster

#### 2.3.1. D. melanogaster Strain and Culture Maintenance

Wild-type *D. melanogaster*, Oregon-R-C strain (Bloomington Drosophila Stock center at Indiana University, USA, strain number 5), was used in this study. The *D. melanogaster* stock culture was cultivated at optimum density and conditions of 25 ± 1 °C, 60% air humidity, and a 12/12-h photoperiod regime. Flies were grown on a standard Drosophila cornmeal-based feeding medium, which contained water, 10% cornmeal, 9% sugar, 2% agar, 2% yeast, and Nipagin fungicide (Alfa Aesar GmbH and Co KG, 76185 Karlsruhe, Germany).

For the life cycle monitoring and oxidative stress assay, fruit flies from the stock culture were set on a standard feeding media for a few hours to lay eggs. Three days thereafter the larvae were collected, rinsed with distilled water, and used for the study.

#### 2.3.2. Experimental Design

Two parallel experiments were conducted in the study. The first experiment evaluated the effects of 6 concentrations of the hemp seed oil (0 (control), 12.5, 18.7, 31.2, 62.5, and 125 μL/mL) compared to a positive control (20 mM of the antioxidant ascorbic acid (ASC)) on the oxidative stress markers and life cycle of *D. melanogaster* (Table 1). The hemp seed oil and the ascorbic acid (vitamin C) were added to the feeding media of the *D. melanogaster* larvae.

The second experiment evaluated the effects of the same 6 concentrations of hemp seed oil (0 (stressed control—S), 12.5, 18.7, 31.2, 62.5, and 125 μL/mL) on the oxidative stress markers and life cycle of *D. melanogaster*, this time under H_2_O_2_-induced oxidative stress conditions, compared to the positive ascorbic acid control (20 mM, ASC + S) and to a non-treated control (w/o the addition of H_2_O_2_ or hemp oil, control-) (Table 1). The oxidative stress was induced by the addition of 0.02% H_2_O_2_ to the feeding medium of the *D. melanogaster* larvae following the transfer to the experimental setup. The concentration of H_2_O_2_ used (0.02%) was determined as a sublethal concentration in a preliminary experiment that monitored *D. melanogaster* survival and life cycle under a range of H_2_O_2_ concentrations (unpublished results). The H_2_O_2_ and ascorbic acid were added to the feeding media of the *D. melanogaster* larvae. The concentrations of the hemp seed oil and the ascorbic acid were selected based on previous studies ([34,35], respectively).

The supplements were added to a freshly prepared liquid feeding medium (at 40 °C) and stirred for homogenization using a magnetic stirrer. Then, 5 mL samples of the treatment medium were poured into 50 mL vials and set to cool down before larval transfer. Next, 100 or 25 larvae for each replicate were transferred to the freshly prepared treatment medium for assessment of treatment effects on the oxidative stress parameters and the life cycle of *D. melanogaster*, respectively.

The range of the oil concentrations to be evaluated in the study was determined by a preliminary study that evaluated oil concentrations and demonstrated similar trends to the current study. The measurements were conducted three times with similar results. All of the measurements were conducted with 3 replicates.

#### 2.3.3. Oxidative Stress Markers

For the evaluation of the effects of hemp seed oil on oxidative stress markers, 100 larvae were transferred into each replicated vial containing a freshly prepared treatment medium. Larvae were randomly chosen and transferred into each vial. The vials were capped with cotton plugs and incubated under optimal laboratory conditions (25 °C, 60% humidity, and 12/12-h day/night regime). Transferred larvae were allowed to feed and incubate under standard laboratory conditions for 72 h. Thereafter, the larvae were washed in buffer saline, weighed, and homogenized in 0.1 M sodium phosphate buffer at pH 7.4 containing 0.15 M KCl. For the evaluation of oxidative stress parameters, 10% (*w*/*v*) larval tissue homogenates were used. The samples were centrifuged at 2300× *g* for 15 min at 4 °C, then the supernatants were used for the assays. The extracts were kept on ice until the analysis. Total protein content in the tissue homogenate was determined by Bradford’s method [36].

##### MDA Assay

Malondialdehyde (MDA) is the end product of lipid peroxidation. MDA formation was determined according to the Ohkawa method [37] with minor modifications. In short, 200 µL of tissue homogenate was mixed with 1.5 mL 20% acetic acid at pH 3.5, with 1.5 mL of 0.8% TBA and 200 µL 8.1 % SDS. The mixture was brought up to the volume of 4 mL with dH_2_O and heated in boiling water for 1 h. After cooling and centrifugation (4000 RPM at 4 °C for 10 min), the MDA content in the supernatant was measured at 532 nm with a UV 1650PC UV-VIS spectrophotometer (Shimadzu Corporation, Tokyo, Japan). MDA levels were determined from the calibration curve and expressed in nmol/mg of larval proteins.

##### Glutathione Assay

Glutathione (GSH) is part of the cell antioxidative system capable of detoxifying reactive oxygen species. Its content in the tissue extract was estimated using Ellman’s reagent, as described by Beutler [38]. In short, the GSH was analyzed in a mixture of 100 µL tissue extract, 200 µL 0.1 % EDTA, and precipitating solution. The extraction was performed on ice in the dark for 15 min and the samples were then centrifuged at 4000 RPM for 10 min. The supernatant (300 µL) was mixed with 750 µL of 0.2 M Na-phosphate buffer at pH 7.4 and 100 µL 5,5′-dithio-bis-2-nitrobenzoic acid (DTNB). The reaction was monitored at 412 nm and the amount of GSH determined from the calibration curve was expressed in nmol/mg of larval protein.

##### Antioxidant Defense Enzymes: Superoxide Dismutase and Catalase

The enzyme superoxide dismutase (SOD) catalyzes the dismutation of the reactive superoxide radical into O_2_ and H_2_O_2_. SOD assay was performed according to the method used by Marklund and Marklund [39], which is based on the inhibition of pyrogallol autooxidation by the SOD enzyme. The reaction mixture contained Tris EDTA buffer at pH 8.2, 0.2 mM pyrogallol, and 50 µL of the enzyme extract. The inhibition was measured spectrophotometrically at 420 nm using a UV 1650PC UV-VIS spectrophotometer (Shimadzu Corporation, Tokyo, Japan), and the SOD activity was expressed as U/mg of protein. One unit of enzymatic activity is the amount of SOD that reduces the autooxidation of pyrogallol by 50%.

The enzyme catalase (CAT) catalyzes the decomposition of H_2_O_2_ to water and molecular oxygen. The catalase assay was conducted according to a standard protocol [40]. The reaction mixture consisted of 250 µL of 0.3% (*v*/*v*) H_2_O_2_, 2.4 mL phosphate buffer (pH 7.0), and 50 µL tissue homogenate. The degradation of H_2_O_2_ by catalase was followed at 240 nm. Catalase activity was expressed as µM of H_2_O_2_ decomposed/min/mg protein.

#### 2.3.4. D. melanogaster Life Cycle

To evaluate the effects of the treatments on the life cycle of *D. melanogaster*, 25 larvae were randomly chosen from each replicate and transferred to a freshly prepared hemp seed oil treatment medium. Replicates were incubated under optimal conditions for tracking key parameters of the fruit fly life cycle, including total numbers of pupated and eclosed units, as well as larval and pupal mortality. Larval mortality was expressed as a percentage of the differences between the total numbers of transferred larvae and formed pupae within the treatment group for each replica. Pupal mortality was expressed as a percentage of the differences between the total numbers of formed pupae and eclosed adults within a treatment group for each replica.

### 2.4. Statistical Analysis

All values are given as means ± standard deviation (SD). The data were analyzed with the SPSS software, version 19.0. Differences among treatments were analyzed using one-way analysis of variance (ANOVA) followed by Tukey’s HSD test. Means were considered significantly different at *p* < 0.05.

## 3. Results

### 3.1. Chemical Analysis of Hemp Seed Oil

The hemp oil was chemically characterized by GC-MS upon transesterification and silylation, as well as by NMR. The ^1^H NMR profile of the oil corresponded to a pure sample of highly unsaturated triacylglycerols, i.e., it did not contain any visible additional constituents. The signals were sufficiently segregated to allow an evaluation of the ratio of saturated, mono-, di-, and triunsaturated fatty acids that are part of the triacylglycerols present in hemp oil. The integration of the signal originating from the methyl group of the α-linolenic acid moiety was the entry point, and the signals of protons in doubly and singly allylic positions were utilized to obtain the desired ratio. The assumption of the presence of only esterified acids was verified by comparing the integrals of protons from the glycerol part with those in the α-position to the ester carbonyl and further to the total integral of the ω-methyl groups. An average triacylglycerol of the hemp seed oil sample contained 0.47 residues of α-linolenic acid, 1.84 residues of linoleic acid, 0.34 residues of oleic acid, and 0.35 residues of saturated acids.

The quantitative NMR results were in agreement with the relative amount of unsaturated fatty acids inferred from the GC-MS analysis of a methanolyzed hemp seed oil sample (Table 2). It was not possible to directly compare the distribution of the polyunsaturated fatty acid moieties due to the coelution of methyl oleate and methyl α-linolenate. However, GC-MS analysis of this transesterified sample enabled a more detailed insight into the exact identities of the present fatty acids; in total, 19 different acids were identified. In addition to the above mentioned fatty acids, a tetraunsaturated acid (stearidonic acid) and γ-linolenic acid, along with other less frequently encountered unsaturated fatty acids, were noted. The saturated fatty acids were dominated by palmitic acid, while the length of the chains ranged from C_14_ to C_24_.

In order to assess the non-triacylglycerol constituents, which were present in minor amounts as evidenced by NMR, a sample of hemp seed oil was silylated and subjected again to GC-MS. These results are presented in Table 3, and it should be kept in mind that the total amount of the listed constituents does not reach more than 0.1%, as inferred from a separate GC-MS run with an internal standard (dodecane) added. The oil was found to contain some free fatty acids, sterols, and two tocopherols. In addition to these, low amounts of a mixture of mono- and diacylglycerols were also detected, but due to coelution, these were not successfully identified (it was evident that they contained expected fatty acid residues). No traces of cannabinoids were found.

### 3.2. Effects of Hemp Seed Oil on Oxidative Stress Markers in D. melanogaster Larvae-Protective Function

**Figure 1:**
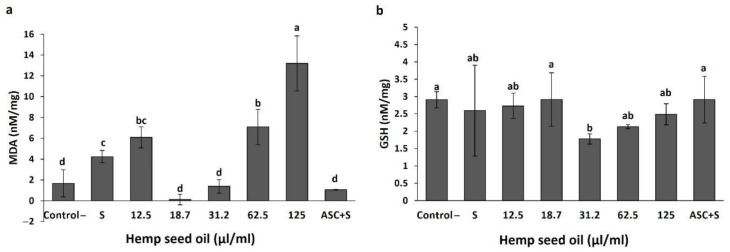
Effects of hemp seed oil on malondialdehyde (MDA) (**a**) and glutathione (GSH) (**b**) contents in *D. melanogaster* larvae. The hemp seed oil was applied to the feeding medium at six concentrations (0 (control), 12.5, 18.7, 31.2, 62.5, and 125 μL/mL) compared to a positive control of the antioxidant ascorbic acid (ASC). The results are means ± SD. Different letters above the means represent significant difference between treatments based on LSD test (*p* < 0 05).

**Figure 2:**
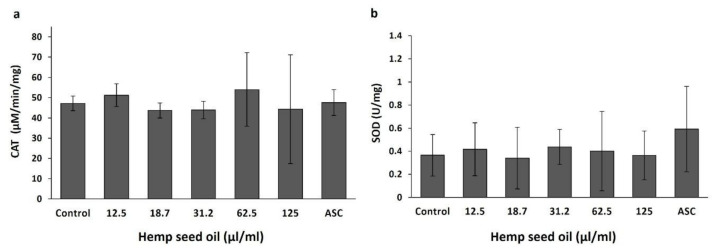
Effects of hemp seed oil on activities of the antioxidative enzymes catalase (CAT) (**a**) and superoxide dismutase (SOD) (**b**) in *D. melanogaster* larvae. The hemp seed oil was applied to the feeding medium at six concentrations (0 (control), 12.5, 18.7, 31.2, 62.5, and 125 μL/mL) compared to a positive control of the antioxidant ascorbic acid (ASC). All values are expressed as means ± SD. There were no statistical differences between treatments (*p* > 0.05, LSD test).

The effects of hemp seed oil on oxidative state parameters under non-stress conditions are presented in Figure 1 and Figure 2. Application of the oil in the concentration range of 12.5–62.5 µL/mL did not significantly affect the malondialdehyde (MDA) content in the larval tissue compared with the non-treated control. A higher oil concentration of 125 µL/mL significantly increased the MDA content (Figure 1a). Ascorbic acid (ASC) decreased the MDA content compared with some of the oil treatments (18.7, 31.2, and 125 µL/mL).

Glutathione (GSH) is an antioxidant capable of detoxifying reactive oxidant species. The treatments affected GSH contents in the larvae (Figure 1b). GSH levels in the larvae, under exposure to 12.5, 62.5, and 125 µL/mL oil, were lower than in the non-treated control (Figure 1b). Vitamin C (ASC), which is a well-known antioxidant and which served as a positive control, also induced a reduction in GSH content in the larvae compared with the non-treated control and the 18.7 µL/mL oil treatment (Figure 1b). Among the oil treatments, exposure to 12.5 and 125 µL/mL oil induced the lowest GSH contents in the larvae (*p* < 0.05, Figure 1b).

Supplementation of hemp seed oil or ascorbic acid to the feeding media did not affect the activity of the antioxidative enzymes catalase (Figure 2a) and superoxide dismutase (Figure 2b) in the *D. melanogaster* larvae.

### 3.3. Antioxidant Activity of Hemp Seed Oil under H2O2-Induced Stress in D. melanogaster Larvae

**Figure 3:**
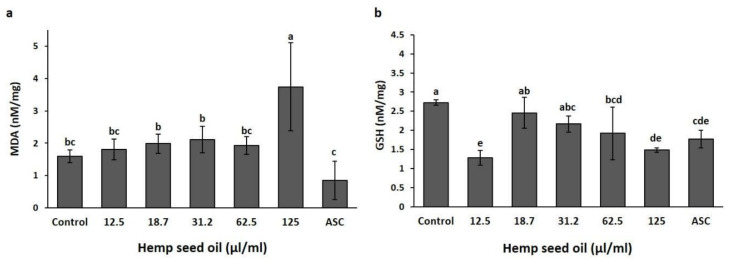
Effects of hemp seed oil on malondialdehyde (MDA) (**a**) and glutathione (GSH) (**b**) contents in *D. melanogaster* larvae under chronic H_2_O_2_-induced oxidative stress. The hemp seed oil was applied to the H_2_O_2_-contained feeding medium at six concentrations (0 (S), 12.5, 18.7, 31.2, 62.5 and 125 μL/mL), compared to an unstressed negative control (control-), and a positive control of ascorbic acid (ASC + S). All values are expressed as means ± SD. Different letters above the means represent significant differences between treatments (*p* < 0 05; LSD test).

**Figure 4:**
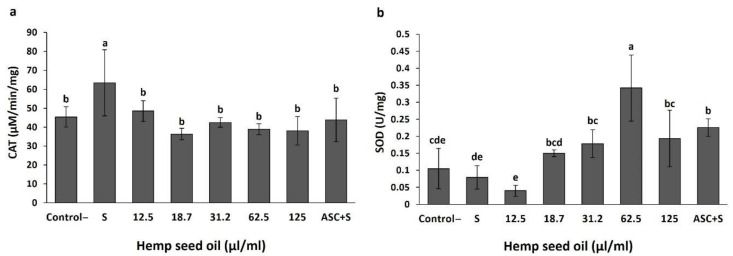
Effects of hemp seed oil on the activity of the antioxidative enzymes catalase (CAT) (**a**) and superoxide dismutase (SOD) (**b**) in *D. melanogaster* larvae under chronic H_2_O_2_-induced oxidative stress. The hemp seed oil was applied to the H_2_O_2_-contained feeding medium at six concentrations (0 (S), 12.5, 18.7, 31.2, 62.5, 125 μL/mL), then compared to an unstressed negative control (control-) and a positive control of ascorbic acid (ASC + S). All values are expressed as means ± SD. Different letters above the means represent significant differences between treatments (*p* < 0.05, LSD test).

The effects of the hemp seed oil on MDA and GSH contents, as well as the activities of catalase and SOD in larval tissue, were evaluated under H_2_O_2_-induced oxidative stress (Figure 3 and Figure 4).

The response of the H_2_O_2_-stressed larval tissue to the hemp seed oil was dose-dependent. Feeding on a nutrient media supplemented with 18.7 or 31.2 μL/mL or with vitamin C maintained MDA contents at levels similar to the non-stressed control (control-), and at significantly lower levels than the concentrations in the non-treated stressed tissue (S). Application of higher oil concentrations of 62.5 and 125 μL/mL did not prevent the increase in MDA under stress but stimulated production of MDA to levels higher than the non-treated stress treatment (S) (Figure 3a). The 125 μL/mL oil treatment elicited production of MDA to levels significantly higher than all other treatments.

GSH levels were not significantly affected by the H_2_O_2_-induced oxidative stress, neither by the application of ascorbic acid under stress (Figure 3b). Application of 31.2 μL/mL oil induced a significant reduction in GSH content compared with the non-stressed control and the ascorbic acid treatment, while higher or lower oil concentrations sustained GSH contents similar to the control and the ascorbic acid treatment (Figure 3b).

The activity of the antioxidant enzyme catalase increased significantly in the larval tissue due to the H_2_O_2_-induced stress (Figure 4a). Under all oil treatments, as well as under the ascorbic acid treatment, the activity of catalase was lower than in the stressed tissue without the addition of the oil and did not differ significantly from the control (Figure 4a).

SOD activity was not affected significantly by the H_2_O_2_-induced oxidative stress (Figure 4b). The response to the oil application was dose-dependent and followed a maximum curve. SOD activity in the stressed tissue was not significantly affected by the lowest oil concentrations (12.5 and 18.7 μL/mL); it increased significantly with further increases in oil concentration up to 62.5 μL/mL and was significantly reduced under higher oil application of 125 μL/mL. The activity of SOD was significantly higher under 31.2–125 μL/mL oil compared with the stress treatment and application of a lower oil dose (12.5 μL/mL). An increase in SOD activity in the stressed tissue was also induced by ascorbic acid. The activity of the enzyme under 62.5 μL/mL oil application was significantly higher than for all other examined treatments (Figure 4b). Although not statistically significant, SOD activity under 18.7 μL/mL oil application was higher compared with the stress treatment and was significantly higher compared to the activity under 12.5 μL/mL oil.

### 3.4. Effects of Hemp Seed Oil on the Life Cycle of D. melanogaster under Non-Stress and H2O2-Induced Oxidative Stress

**Figure 5:**
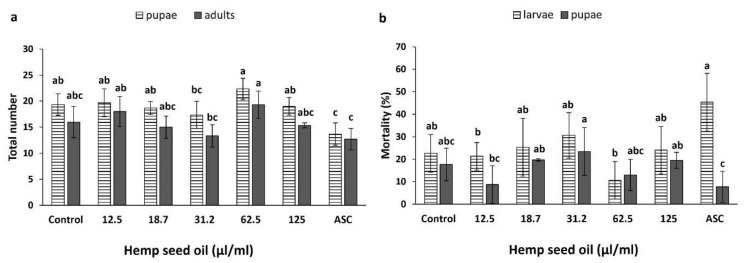
Effects of hemp seed oil and ascorbic acid on the total numbers of formed pupae and eclosed adults (**a**) and percentages of larval and pupal mortality (**b**). The larvae were exposed to six concentrations of the oil (0 (control), 12.5, 18.7, 31.2, 62.5, and 125 μL/mL) and compared to a positive control of the antioxidant ascorbic acid (ASC). All values are expressed as means ± SD. Different letters above the means represent significant differences between treatments separately for larvae, pupae, or eclosed adults (*p* < 0 05; LSD test).

Under non-stress conditions, the oil treatments did not significantly affect the total number of formed pupae (Figure 5a); however, ascorbic acid significantly reduced the total number of pupae compared with the control and most oil treatments (excluding the 31.2 μL/mL treatment). With 31.2 μL/mL oil concentration, the population was significantly smaller than with the 62.5 μL/mL concentration.

The number of eclosed adults (flies) was not affected significantly by any of the oil or ascorbic acid treatments (Figure 5a). The results for the total number of adults were similar to the results for the pupae. Although there was no significant difference between the control and the oil treatments, the numbers of eclosed adults recorded for the 12.5 and 62.5 μL/mL oil concentrations were significantly higher than for the ascorbic acid treatment.

The percentages of larval and pupal mortality were not affected significantly by any of the oil or the ascorbic acid treatments compared with the control (Figure 5b). The lowest percentage of larval mortality (10.33%) and the largest difference from the positive ascorbic acid control were recorded for the 62.5 μL/mL hemp oil treatment. The ascorbic acid treatment also showed a significantly higher larval mortality compared with the 12.5 and 62.5 μL/mL treatments (Figure 5b). Pupal mortality increased with the increase in hemp seed oil concentration up to 31.2 μL/mL (Figure 5b). It was significantly higher under 18.7, 31.2, and 125 μL/mL compared with the ascorbic acid positive control. Pupae from the 12.5 μL/mL treatment showed significantly lower mortality than from the 31.2 μL/mL oil treatment (Figure 5b).

**Figure 6:**
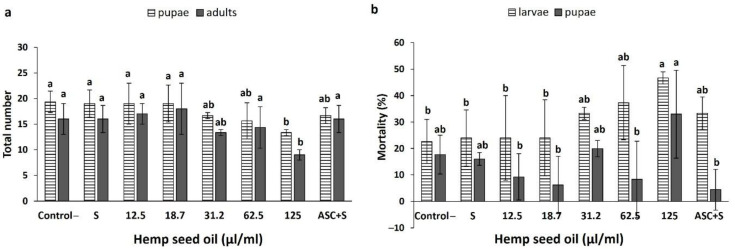
Effects of hemp seed oil and ascorbic acid on the total numbers of formed pupae and eclosed adults (**a**) and percentages of larval and pupal mortality (**b**) under chronic H_2_O_2_-induced oxidative stress. The hemp seed oil was applied to the H_2_O_2_-contained feeding medium at six concentrations (0 (S), 12.5, 18.7, 31.2, 62.5, and 125 μL/mL) and compared to an unstressed negative control (control-) and a positive control of ascorbic acid (ASC + S). All values are expressed as means ± SD. Different letters above the means represent significant differences between treatments separately for larvae, pupae, or eclosed adults (*p* < 0.05; LSD test).

The effects of the hemp seed oil application in the larval feeding medium on the life cycle of D. *melanogaster* under chronic oxidative stress conditions are presented in Figure 6. The H_2_O_2_-induced stress did not affect the numbers of formed pupae or eclosed files (Figure 6a). Hemp seed oil in the concentrations range of 12.5–62.5 μL/mL, as well as ascorbic acid, did not affect the total numbers of pupae or files (Figure 6a). The highest concentration of the oil investigated, 125 μL/mL, significantly reduced the numbers of pupae and files, demonstrating a toxicity response under oxidative stress condition.

The H_2_O_2_-induced stress also did not affect the larval or pupal mortality (Figure 6b). Similar to the effects of the oil on the total numbers of formed pupae and eclosed files, the highest concentration of oil tested (125 μL/mL) demonstrated toxicity effects. It induced increases in mortality that reached 46.67% and 32.97% for the larvae and pupae, respectively (Figure 6b). Lower concentrations of the oil, as well as ascorbic acid, did not significantly affect the percentages of larval and pupal mortality, which were maintained at similar levels to the control.

## 4. Discussion

Due to the high nutritional value and the unique combination of polyunsaturated fatty acids and antioxidants of hemp seed, its consumption is considered to improve the overall condition of the organism and to improve various disease conditions. Hemp seed preparations have been demonstrated to have antioxidant properties in vitro [41,42,43,44,45] and antioxidant activity in vivo [46]. Hemp seed oil is also known for its antioxidant potential [22,28]. Despite the potential benefits, only a few animal studies have evaluated the effects of hemp seed oil at the organism level. More information is, therefore, needed on the impacts on the cell redox status in vivo when the oil is used for preventive measures under non-stress conditions, as well as the possibility for reducing oxidative stress under chronic oxidative stress conditions.

The present study examined the effects of various hemp seed oil concentrations on the cell oxidative status of healthy animals, with the goal of assessing the possibility of boosting the antioxidative defense system towards use as a preventative tool. Additionally, the effects of the oil on the oxidative status were also evaluated under chronic (H_2_O_2_-induced) oxidative stress conditions to evaluate the potential for therapeutic use under chronic oxidative stress. The impacts of the oil on the life cycle of *Drosophila* were analyzed to provide an integrative view of the organism following larvae exposure to the treatments.

Hemp seed oil is known to contain various plant secondary metabolites that maintain the oxidative stability of the oil by preventing oxidation of unsaturated fatty acids. Tocopherols, especially γ-isoform, have been recognized to have the highest antioxidant potential in hempseed oil, although phenolic compounds may also play a significant role as antioxidants [47]. The antioxidants found in the extracted hemp seed oil used in the study were small amounts of tocopherols (γ and α). Additionally, 88.3% of unsaturated fatty acids (15.7%, 61.3%, and 11.3% for tri-, di-, and monounsaturated fatty acids, respectively), while 11.6% of their saturated counterparts were identified in the oil. The ratio between ω-6 and ω-3 was around 3.9, which is among the lowest values known for such oils [48]. The fatty acid profile of hemp seed oil often contains predominantly linoleic acid, followed by α and γ-linolenic acids [49].

As an animal model, we used *D. melanogaster,* a widely used model organism for the study of the effects of pharmacological compounds on diseases, due to the short life cycle and lifespan and the similarity of signaling pathways and genes related to diseases between *Drosophila* and humans [50,51,52]. *Drosophila* is also a suitable model organism for testing the antioxidant potential of plant components [50]. Furthermore, longevity in *Drosophila* is associated with high levels of antioxidant enzyme activity [53].

Under non-stress conditions, a significant effect of the hemp seed oil on MDA content was induced only by the highest application amount (125 μL/mL, Figure 1a). This indicates that this concentration is not suitable as a preventive treatment because it leads to a higher level of lipid peroxidation and membrane disruption. Many studies have demonstrated a correlation between the content of polyunsaturated fatty acids and the extent of lipid peroxides [33,54].

Two concentrations of the oil, 18.7 and 31.2 μL/mL, did not reduce the GSH level compared with the control (Figure 1b). These concentrations maintain the antioxidative potential of the cell by maintaining GSH levels similar to the control. This indicates that the mechanism for effects on the cell redox status under exposure to these concentrations varies from the mode of action of ascorbic acid (a well-known antioxidant), which led to reduced GSH levels compared to the control. Shang et al. [55] reported that the protective effects of vitamins C (ascorbic acid) are not associated with restoration of GSH levels in GSH-depleted cells, since GSH was lower in the ascorbic acid treatment than in the control. They explained that the antioxidative effect of vitamin C is associated with enhanced redox status (higher GSH:GSSG ratio), as is evident from the decreased concentration of oxidized glutathione in vitamin-C-supplemented cells compared with control cells.

Hemp seed oil, similar to 20 mM ascorbic acid, did not significantly affect the activities of the antioxidative enzymes catalase and SOD in *Drosophila* under non-stress conditions. This indicates that preventive intake of the oil or ascorbic acid did not change the enzymes’ antioxidative defense activity. Valls et al. [56] showed a reduction in catalase activity after the application of corn oil in Wistar rats. They also reported no difference in SOD activity under a hyperlipidic diet.

For the in vivo evaluation of the antioxidative potential of hemp seed oil under oxidative-stress conditions, we chose H_2_O_2_ as a stress inducer. Hydrogen peroxide is a reactive oxygen molecule, the product of the antioxidant’s reactions, and a signal molecule in cells [16]. Its ability to induce oxidative stress has been shown before in acute and chronic treatments [35,57,58,59].

MDA, as the product of lipid peroxidation that causes the disruption of cell membranes, is a reliable indicator of oxidative stress. Stress conditions increased MDA content in larval tissues as compared to the non-stress negative control (Figure 3a), indicating stimulation of lipid peroxidation by the stress. Exposure to 0.02% H_2_O_2_ did not affect the mortality of the larvae, as was identified by the lack of effects on the numbers of pupae and flies (Figure 6). Taken together with the MDA content results, this establishes our model system of study as a suitable model for analyzing the effects of hemp seed oil under oxidative stress conditions. Under a chronic treatment with H_2_O_2_ alone, catalase activity increased significantly, while SOD activity was not significantly affected. These results are in accordance with the results of previous studies, which showed similar effects of an acute H_2_O_2_-induced stress on these enzyme activities [35] and in the response of catalase activity [60].

The highest concentration of the hemp seed oil proved unfavorable under stress. It stimulated lipid peroxidation in the larvae, as was apparent by the increase in MDA, and increased the mortality rates of pupae and flies (Figure 3 and Figure 6). Increases in the numbers of dead pupae and flies under application of high oil concentrations occurred only under stress conditions. Under non-stress conditions, high oil concentrations did not affect pupa or fly numbers, although the content of MDA increased (Figure 1 and Figure 5). By adding hydrogen peroxide, the organism needed to cope with two different sources of stress simultaneously, namely H_2_O_2_ and the high amount of unstable polyunsaturated fatty acids from the oil that may undergo peroxidation. Polyunsaturated fatty acid treatments were demonstrated to induce different effects on MDA content [33,61]. In accordance with these trends, our results demonstrate that the type of response, being either an increase or decrease in MDA content under chronic stress, is dependent on the oil concentration. Under H_2_O_2_-induced stress conditions, SOD activity was also dose-dependent. This is unlike the activity under non-stressed conditions, which was not affected by the oil concentration alone. Increased activity of the antioxidative enzymes SOD and catalase is related to the effectiveness of the defense against different stress inducers, including H_2_O_2_ [62,63,64]. Hong et al. [43] reported that hemp seed extracts increased gene expression of antioxidant enzymes (SOD, glutathione peroxidase, and catalase) in a concentration- dependent manner in H_2_O_2_-challenged HepG2 cells, indicating their influence on the antioxidative enzyme system and not only on the scavenging potentials.

Hemp oil concentrations of 18.7 and 31.2 μL/mL increased SOD activity, with a favorable outcome, while further increases in oil concentrations and SOD activity had no healing effect, as confirmed by the increased amount of MDA. Therefore, the larvae were capable of coping with the stress under the 18.7 and 31.2 μL/mL oil treatments, which maintained MDA content in the larvae at levels similar to non-stressed conditions (control-) and the ascorbic acid treatment. This indicates the effects of the oil and of ascorbic acid on the reduction of lipid peroxidation and stress. Under stress conditions, the lowest oil concentration did not reduce stress, as was demonstrated by the amount of MDA produced, nor did it increase SOD activity. The effects of hemp seed oil on the antioxidative system had not been investigated until now. The obtained effects under induced oxidative stress are in accordance with previous reports for other seed oils, confirming antioxidative function in spite of the high amount of polyunsaturated fatty acids [56,61].

Application of hemp seed oil under oxidative stress conditions reduced the activity of catalase to the level of the non-stressed cells (Figure 4). H_2_O_2_ is an endogenous molecule that is well regulated in mammalian cells by the catalase and glutathione peroxidase. However, *Drosophila* lack GR and Gpx activity, and the Gpx gene (known as a glutathione peroxidase homolog), which has a thioredoxin peroxidase activity (GTpx-1), has an antioxidant function that cannot compensate for the lack of catalase activity [65]. Since its role is irreplaceable in *Drosophila*, we assume that the reduction of catalase levels to the control levels by the oil application treatments resulted from the oil contribution to maintaining H_2_O_2_ levels by stimulating its faster removal or from other mechanisms that act in a catalase-independent manner [65,66,67].

In *Drosophila* larvae, GSH serves as an intracellular antioxidant and also plays a role in ecdysteroid biosynthesis in early *D. melanogaster* larval development [68]. In our study, treatments with 31.2 and 18.7 μL/mL concentrations of hemp seed oil acted differently on GSH levels, but with the same outcome. GSH content was statistically lower after a treatment with 31.2 μL/mL compared with 18.7 μL/mL oil, but both concentrations decreased MDA content, demonstrating that they reduced lipid peroxidation and stress. Under treatment with 31.2 μL/mL of oil, there was an increased need for GSH to successfully maintain the oxidative cell status. Under other oil treatments, which showed GSH values similar to the untreated control, MDA content was not lower than in the stressed treatment. As an antioxidant, GSH is involved in mechanisms of detoxification by conjugation, via its free radical scavenging activity and metabolism of various compounds, indicating its significant crucial role in the antioxidant defense machinery [69].

Taken together, the results indicate that 31.2 μL/mL is the threshold concentration suitable for providing a healing effect. Higher oil concentrations of 62.5 and 125 μL/mL induced increases in MDA content and SOD activity, which was higher with the 62.5 μL/mL treatment, as well as mortality under 125 μL/mL. These concentrations were not effective under H_2_O_2_-induced stress and are not suitable for the treatment of oxidative stress-associated conditions. Surprisingly, the lowest oil concentration induced an increase of MDA, indicating that the concentration of 12.5 μL/mL was insufficient to boost cell antioxidative defense under stress conditions, however the antioxidant function of the oil probably prevented mortality. The optimal antioxidative results were obtained with 18.7 μL/mL oil application.

The dose-dependent antioxidant effect of the hemp seed oil and the lack of effect at the lowest applied oil concentration (12. 5 mg/mL) indicate that the antioxidant potency of the oil is correlated to its chemical composition. The absolute amount of the detected tocopherols does not seem to be sufficient to warrant the oil a significant antioxidant properties. However, the very high unsaturated nature of the fatty acids identified makes the oil a quencher of reactive radicals due to numerous doubly allylic methylene groups, leading to resonance-stabilized radicals and most probably a polymerization of the oil [70]. It has been reported that linoleic acid does not have radical-quenching abilities, as determined in vitro by photoemission, electron spin resonance spectrometry, and spectrophotometric method with DPPH•, whereas conjugated linoleic acid isomers do have radical-quenching abilities [71,72]. In vivo, the antioxidant activity of polyunsaturated fatty acids and their susceptibility to lipoperoxidation do not have to be directly correlated with chain length or degree of saturation [73]. Oleic, linoleic, arachidonic (20:4n), eicosapentaenoic (EPA-20:5n), and docosahexaenoic (DHA-22:6n) acids scavenged the superoxide anion in human aortic endothelial cells, while linoleic acid and its conjugated isomers showed better antioxidant capacity than α-linolenic and γ-linolenic acids in bovine mammary epithelial cells under H_2_O_2_-induced stress conditions [74,75]. Vegetable seed oils rich in α-linolenic acid can significantly improve the antioxidant status in rats [76]. In mammalian cells, α-linoleic acid is converted to conjugated ω-3 isomers (DHA, EPA, and docosapentaenoic acid-DPA-22:5n), while linoleic acid via γ-linolenic acid is metabolized to arachidonic acid and eicosadienoic acid (20:2n-6) through the other pathway [77]. DHA and EPA can activate the Keap1/Nrf2 signaling pathway, the major route triggering antioxidant cell response, via stimulation of Nrf2 transcription factor [78,79]. An oxidized metabolite of linoleic acid (12,13-epoxy-9-keto- 10(trans)-octadecenoic acid (EKODE)) initiated antioxidant response in neuroblastoma cells and cerebrocortical neurons via activation of the antioxidant response element (ARE), the expression of which is governed by the Nrf2 transcription factor [78]. Additionally, low lipoperoxidation can slightly increase the ROS concentration, leading to activation of the Nrf2 pathway [80]. Keap1/Nrf2 signaling is conserved through evolution, and in the fruit fly it can be activated by oxidants, antioxidants, and detoxification responses [81].

Data on antioxidant activities of seed oils or polyunsaturated fatty acids in *Drosophila* models are very limited and restricted to the studies on adults. Feeding fruit flies stressed by H_2_O_2_ with hemp seed or linoleic acid demonstrated that both hemp seed and linoleic acid had antioxidant capacity and that linoleic acid was partly responsible for this activity of the hemp seed [82]. Borage seed oil and linolenic acid, its major constituent, together with linolenic acid, prevented H_2_O_2_-induced DNA damage [34]. EPA and DHA inhibited lipoperoxidation in fruit flies and showed protective effects against oxidative damage in older flies, but not at younger ages, due to higher SOD activity [83]. Diet supplementation with EPA and DHA protected *Drosophila* against paraquat toxication by restoring mitochondrial functions and decreasing H_2_O_2_ production [84].

Linoleic and α-linolenic acids are essential for *D. melanogaster*, especially for larvae, who prefer unsaturated fatty acids in the diet, in contrast to adults, who prefer saturated fatty acids [85]. In *Drosophila*, linoleic and α-linolenic acids cannot be transformed to polyunsaturated fatty acids with more than 20 carbon atoms due to the deficiency in genes encoding Δ5 and Δ6 desaturases [86], indicating that lipid signal mediators similar to those in mammalian cells cannot be responsible for the antioxidant activity. The recent discovery of the lipid signal mediators 2-linoleoyl glycerol, 2-oleoyl glycerol, and 45 N-acyl amides in *Drosophila* larval tissues suggests that in the fruit fly, lipid endocannabinoid signaling exists in an alternative pathway compared to mammalian cells [87]. Therefore, it should not be ruled out that there are lipid signaling molecules—activators of antioxidant cell response in *Drosophila*—that are different from those in mammalian cells. Huangfu et al. [88], after evaluating the effects of feeding fruit flies with marine microalgae rich in DHA, concluded that DHA probably achieve effects by binding to ligands connected to transcription factors rather than by interactions with ROS.

Another way in which polyunsaturated fatty acids could have an antioxidant activity is by changing the chemical composition of the cell membranes. Dietary fatty acids influence lipid packing in membranes, including mitochondrial membranes, leading to modulation of ROS generation through lipid peroxidation. Different fatty acid diets resulted in different lipid profiles in *Drosophila* larvae, and adults and larvae fed with polyunsaturated fatty acids showed better mitochondrial respiratory capacity under cold stress [89]. Copper-induced toxicity, which increased the incorporation of saturated fatty acids in fish cell membranes, was reverted by application of dietary hemp seed oil, leading to a balanced ω-3/ω-6 ratio [31]. In bovine mammary epithelial cells under H_2_O_2_-induced stress, linoleic, α-linolenic, and γ-linolenic acids strongly suppressed lipoperoxidation, probably via the formation of a low concentration of ROS, which can trigger an antioxidant defense [75].

## 5. Conclusions

Under non-stress conditions, hemp seed oil at concentrations up to 62.5 μL/mL (specifically 18.7 and 31.2 μL/mL) did not induce toxic effects on the biological and biochemical parameters in *D. melanogaster* larvae and maintained the redox status of the larvae cells at similar levels to the control level. Under oxidative stress conditions, caution should be exercised with oil concentrations. For *Drosophila*, 18.7 and 31.2 μL/mL hemp seed oil exerted beneficial effects on the oxidative stress markers and also contributed to a successful defense against stress, probably due to the high level of polyunsaturated fatty acids (mainly linoleic and α-linolenic acids) in the oil. However, higher oil concentration (125 μL/mL) exerted negative effects on the oxidative status and increased larval mortality. The presented results reveal that hemp seed oil is effective at defined concentrations for reducing oxidative stress at the cellular level, which points to the potential for the prevention or treatment of diseases caused by the action of oxygen reactive species.

## Figures and Tables

**Table 1:** The experimental treatments evaluated in the study. Ascorbic acid (ASC) was added to the feeding medium of the *D. melanogaster* larvae at the concentration of 20 mM. H_2_O_2_ was added to the feeding medium in sublethal concentrations (0.02%).

Experiment 1 (Non-Stress Conditions)	Experiment 2 (H_2_O_2_-Induced Oxidative Stress Conditions)
Hemp seed oil (μL/mL)	Hemp seed oil (μL/mL)
-	0-H_2_O_2_ (unstressed negative control; (control-))
0 (control)	0 (stressed control; (S))
12.5	12.5
18.7	18.7
31.2	31.2
62.5	62.5
125	125
0 + ASC (positive control, (ASC))	0+ ASC (positive control; )ASC + S))

**Table 2:** The relative amounts of fatty acid methyl esters obtained by methanolysis of *C. sativa* seed oil.

RI	Rel. Amount (%)	Compound
1724	tr	Methyl tetradecanoate (methyl myristate)
1824	tr	Methyl pentadecanoate
1895	0.1	Methyl (9*Z*)-9-hexadecenoate (methyl palmitoleate)
1924	7.6	Methyl hexadecanoate (methyl palmitate)
2014	tr	Methyl (9*Z*)-9-heptadecenoate
2024	tr	Methyl heptadecanoate (methyl margarate)
2083	3.2	Methyl (6*Z*,9*Z*,12*Z*)-6,9,12-octadecatrienoate (methyl γ-linolenate)
2090	1.0	Methyl (6*Z*,9*Z*,12*Z*,15*Z*)-6,9,12,15-octadecatetraenoate (methyl stearidonate)
2108	54.5	Methyl (9*Z*,12*Z*)-9,12-octadecadienoate (methyl linoleate)
2116	27.6	Methyl (9*Z*)-9-octadecenoate (methyl oleate) *
2116		Methyl (9*Z*,12*Z*,15*Z*)-octadeca-9,12,15-trienoate (methyl α-linolenate) *
2124	3.6	Methyl octadecanoate (methyl stearate)
2311	0.7	Methyl (11*Z*)-11-eicosenoate
2324	1.1	Methyl eicosanoate (methyl arachate)
2424	tr	Methyl heneicosanoate
2506	tr	Methyl (13*Z*,16*Z*)-13,16-docosadienoate
2524	0.4	Methyl docosanoate (methyl behenate)
2624	tr	Methyl tricosanoate
2724	0.2	Methyl tetracosanoate (methyl lignocerate)
	100	Total
	87.1	Unsaturated
	12.9	Saturated

RI—experimentally determined retention index based on a homologous series of *n*-alkanes (C_20_-C_40_); tr—trace elements. * Compounds coeluted under the utilized chromatographic conditions.

**Table 3:** Composition of the GC-MS-analyzable silylated *C. sativa* seed oil.

RI	Rel. Amount (%)	Compound
2050	6.4	Palmitic acid, trimethylsilyl ester
2214	18.1	Linoleic acid, trimethylsilyl ester
2229	11.6	Oleic acid, trimethylsilyl ester
2255	0.9	Octadecanoic acid, trimethylsilyl ester
3014	13.2	γ-Tocopherol, trimethylsilyl ether
3136	1.1	α-Tocopherol, trimethylsilyl ether
3263	7.9	Campesterol, trimethylsilyl ether
3286	2.3	Stigmasterol, trimethylsilyl ether
3344	29.7	β-Sitosterol, trimethylsilyl ether
3359	5.7	(24Z)-3-[(Trimethylsilyl)oxy]stigmasta-5,24(28)-diene
	97.0	Total

RI—experimentally determined retention index based on a homologous series of *n*-alkanes (C_20_-C_40_).

## Data Availability

The data are contained within the article.
